# Adolescent Health Interventions: Conclusions, Evidence Gaps, and Research Priorities

**DOI:** 10.1016/j.jadohealth.2016.05.006

**Published:** 2016-10

**Authors:** Rehana A. Salam, Jai K. Das, Zohra S. Lassi, Zulfiqar A. Bhutta

**Affiliations:** a Division of Women and Child Health, Aga Khan University, Karachi, Pakistan; b Robinson Research Institute, University of Adelaide, Adelaide, Australia; c Centre for Global Child Health, The Hospital for Sick Children, Toronto, Ontario, Canada; d Center of Excellence in Women and Child Health, The Aga Khan University, Karachi, Pakistan

**Keywords:** Adolescent health, Adolescent sexual health, Substance abuse, Mental health, Adolescent nutrition, Adolescent immunization, Injury prevention

## Abstract

Adolescent health care is challenging compared to that of children and adults, due to their rapidly evolving physical, intellectual, and emotional development. This paper is the concluding paper for a series of reviews to evaluate the effectiveness of interventions for improving adolescent health and well-being. In this paper, we summarize the evidence evaluated in the previous papers and suggest areas where there is enough existing evidence to recommend implementation and areas where further research is needed to reach consensus. Potentially effective interventions for adolescent health and well-being include interventions for adolescent sexual and reproductive health, micronutrient supplementation, nutrition interventions for pregnant adolescents, interventions to improve vaccine uptake among adolescents, and interventions for substance abuse. Majority of the evidence for improving immunization coverage, substance abuse, mental health, and accidents and injury prevention comes from high-income countries. Future studies should specifically be targeted toward the low- and middle-income countries with long term follow-up and standardized and validated measurement instruments to maximize comparability of results. Assessment of effects by gender and socioeconomic status is also important as there may be differences in the effectiveness of certain interventions. It is also important to recognize ideal delivery platforms that can augment the coverage of proven adolescent health–specific interventions and provide an opportunity to reach hard-to-reach and disadvantaged population groups.

Adolescent health care is challenging compared to that of children and adults, due to their rapidly evolving physical, intellectual, and emotional development [1], [2]. Evidence from high-income countries as well as low- and middle-income countries (LMICs) suggests that services targeting adolescents are highly fragmented, poorly coordinated, and uneven in quality [3]. Furthermore, health practitioners face several challenges with adolescents as they require specialized skills for consultation, interpersonal communication, and interdisciplinary care. This paper is a concluding paper for a series of reviews conducted to evaluate the effectiveness of interventions for improving adolescent health and well-being. Previous seven paper focused on the background, methodology, and conceptual framework [4]; interventions for adolescent sexual and reproductive health [5]; interventions to promote adolescent nutrition [6]; interventions to improve access and coverage of adolescent immunizations [7]; interventions to prevent substance abuse [8]; interventions for adolescent mental health and violence prevention [9]; and interventions to prevent accidents and unintentional injuries among adolescents [10]. Our aim was to look at the holistic evidence around the interventions identified in our conceptual framework for which we took a systematic approach to consolidate the existing evidence through three methodologies: overview of systematic reviews, updating existing reviews, and conducting de novo reviews where no reviews existed, the details of which are described in a separate paper [4]. In this paper, we summarize the evidence evaluated in the previous papers and suggest areas where there is enough existing evidence to recommend implementation and areas where further research is needed to reach consensus.

## Evidence Summary

• Our review findings suggest that interventions for adolescent sexual and reproductive health including education, counseling, and contraceptive provision are effective in increasing sexual knowledge, contraceptive use, and decreasing adolescent pregnancy. Among interventions to prevent female genital mutilation/cutting, community mobilization and female empowerment strategies have the potential to raise awareness of the adverse health consequences of female genital mutilation/cutting and reduce its prevalence; however, there is a need to conduct methodologically rigorous intervention evaluations. There was limited and inconclusive evidence for the effectiveness of interventions to prevent intimate partner violence [5].
• Review on adolescent nutrition interventions suggests that micronutrient supplementation among adolescents (predominantly females) can significantly decrease anemia prevalence, while interventions to improve nutritional status among ‘pregnant adolescents’ significantly improved birth weight and decreased low birth weight and preterm delivery. Interventions to promote nutrition and prevent obesity had a marginal impact on body mass index (BMI) [6].
• Evidence on interventions to improve immunization uptake suggested an overall increase in vaccination coverage through implementing vaccination requirement in school and sending reminders and national permissive recommendation for adolescent vaccination. Interventions to improve vaccine coverage also led to significant declines in the prevalence of human papillomavirus, genital warts, varicella deaths, measles incidence, rubella susceptibility, and incidence of pertussis; however, the data are from very limited and low-quality studies [7].
• Evidence on substance abuse suggest that among smoking/tobacco interventions; school based prevention programs and family based intensive interventions typically addressing family functioning are effective in reducing smoking, mass media campaigns are also effective given that these were of reasonable intensity over extensive periods of time. Among interventions for alcohol use; school based alcohol prevention interventions have been associated with reduced frequency of drinking, family based interventions have a small but persistent effect on alcohol misuse among adolescents. For drug abuse; school based interventions based on a combination of social competence and social influence approaches have shown protective effects against drugs and cannabis use. Among the interventions targeting combined substance abuse; school based primary prevention programs are effective. Evidence from internet based interventions, policy initiatives and incentives appears to be mixed and needs further research. [8].
• Evidence from school based mental health interventions suggest that targeted group-based interventions and cognitive behavioral therapy (CBT) were found to be effective in reducing depressive symptoms and anxiety. School based suicide prevention programs suggest that classroom-based didactic and experiential programs increased short-term knowledge of suicide and knowledge of suicide prevention with no evidence of an effect on suicide-related attitudes or behaviors. Community based creative activities had some positive effect on behavioral changes, self-confidence, self-esteem, levels of knowledge and physical activity. Evidence from digital platforms supports internet-based prevention and treatment programs for anxiety and depression. Among individual and family based interventions; interventions focusing on eating attitudes and behaviors showed no impact on BMI; eating attitude test (EAT); and bulimia. Exercise was found to be effective in improving self-esteem and reducing depression score with no impact on anxiety scores [9].
• Among interventions to prevent unintentional injuries, graduated driver license (GDL) significantly reduced road accidents. There was no impact of GDL programs on incidence on injuries, helmet use and seatbelt use. Sports-related injury prevention interventions led to reductions in the incidence of injuries, incidence of injury per hour of exposure and injuries per number of exposures. Subgroup analysis according to the type of interventions suggests that training ± education and the use of safety equipment had significant impacts on reducing the incidence of injuries [10].

The impact estimates for all interventions reviewed are summarized in Table 1.

## Data Gaps

Most of the outcomes were rated as low or moderate in methodological quality due to lack of rigorous study designs as many of the studies used before-after designs without comparable controls. Trial designs also continued to be compromised by nonrandom allocations as randomization and allocation concealment was not always possible due to the nature of the intervention. Many of the studies focusing on behavior change interventions did not use standardized outcome measures and hence could not be pooled. Many studies also had short follow-up duration. Since majority of the behavior change and psychosocial interventions require a longer duration to achieve an impact, they might not have been able to capture the actual impact. There was lack of evidence on marginalized populations and also on differences of effects according to gender. Most of studies for improving immunization coverage, substance abuse, mental health, and accidents and injury prevention have been completed predominately in high-income countries, and although there is evidence on a more multicultural population in these countries, specific impacts on these disadvantaged populations could not be drawn.

## Implications for Future Research

Future studies should specifically be targeted toward the LMIC to evaluate the effectiveness of adolescent health interventions in these settings. Further studies with longer term follow-ups are required, and study authors should use standardized and validated measurement instruments to maximize comparability of results. Assessment of effects by gender and socioeconomic status is important, and future studies should also take this into account, as there may be differences for certain interventions and this information would be valuable. As adolescent health is still an evolving area with many of their needs unmet, it would be important to carry out an exercise involving experts of adolescent health to prioritize research gaps and recommend immediate areas of action. In addition, to identify further gaps in evidence for adolescent health, this exercise can provide donors with a comprehensive view of projected importance and feasibility of investing in these research gaps along with an idea of the relative importance of the each research priority.

**Figure 1:**
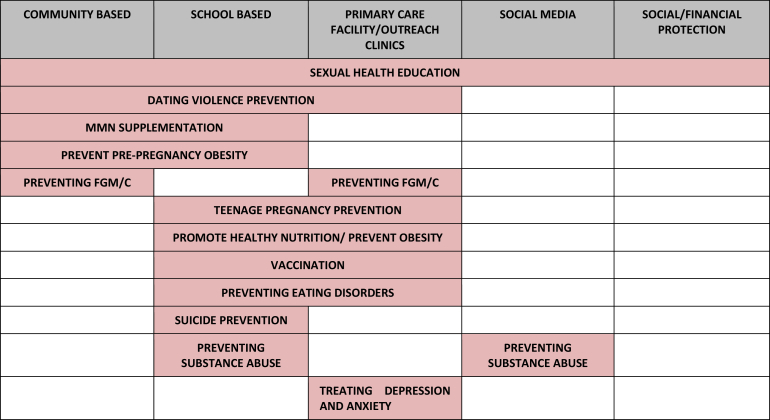
Existing evidence of adolescent health–specific interventions according to the delivery platforms utilized. FGM/C = female genital mutilation/cutting; MMN = multiple micronutrient supplementation.

It is also important to recognize ideal delivery platforms that can augment the coverage of proven adolescent health–specific interventions and provide an opportunity to reach hard-to-reach and disadvantaged population groups. Figure 1 highlights the delivery platforms utilized for the various interventions reviewed in this series of papers. These platforms include school- and community-based delivery, use of communication and information technology, specialized health services (like clinics, health posts, health centers, and district hospitals), youth organizations, and financial incentives. These existing platforms could be utilized to make services “adolescent friendly,” that is, these should be equipped to systematically respond to the barriers to service use that adolescents and service providers have identified. Within each platform, the focus, content, and organization of the services can vary. Existing evidence suggests that school-based programs have been utilized for improving knowledge of sexual abuse and self-protective behaviors [11], prevention of tobacco use [12], [13], reducing aggressive behavior [14], nutrition education interventions, and physical activity programs [15], [16]. However, there is no existing evidence to support the effectiveness of formulating and implementing policies aiming to prevent smoking initiation or improving nutrition in schools [17], [18]. Community-based delivery platforms have been widely utilized for the promotion of maternal, newborn, and child health and are now widely recognized as an important strategy to deliver key maternal and child survival interventions and to reduce inequities [19], [20]. These platforms can also be used to target adolescents to improve their health. In recent years, communication, information technology, and mass media have rapidly evolved into a platform that provides innovative opportunities for engaging youth, including disadvantaged and hard-to-reach youth and those turned off by traditional health education approaches [21], [22], [23], [24], [25], [26]. Despite widespread emphasis on youth centers as a strategy for encouraging young people to access sexual and reproductive health services, results from these studies have not been encouraging, and cost-effectiveness is likely to be low [27]. There is very limited and inconclusive evidence on effects of youth empowerment programs outside of formal education [28], [29], [30].

The World Health Organization and the United Nations Programme on HIV and AIDS have recently released global standards for quality health care services for adolescents to assist policy makers and health service planners in improving the quality of health care services so that the adolescents find it easier to obtain the health services that they need to promote, protect, and improve their health and well-being. These series, based on four volumes, focus on standards and criteria; implementation guide; tools to collect data; and scoring sheets for data analysis [3], [31], [32], [33].

## Recommendations and Conclusions

Compromised adolescent health will negatively affect a country's economy, which will be more pronounced in LMICs. Failure to invest in the health care of adolescents will further increase in the number of dependents in coming generations and negatively influence the health of future generations. It is therefore imperative to work toward improving adolescent health in order to ensure a brighter future for coming generations. Sustainable development goals provide an opportunity for renewed attention to meeting the health care needs of adolescents through the strengthening of health systems. This requires a specific focus on modes and channels of delivering targeted interventions via specialized health services (such as clinics, health posts, health centers, and district hospitals), school-based delivery, youth organizations, community-based delivery, information communication technology, and mass media. To make progress toward universal health coverage, ministries of health and the health sector more generally will need to transform how health systems respond to the health needs of adolescents. A number of transitions in service delivery, workforce capacity, and financing will be needed. Three types of interventions have been stated to be required for increasing the utilization of services by adolescents—some changes in the health facilities (or spot of service delivery), some changes in the attitudes of providers, and sensitization of the community gatekeepers (such as parents, teachers, and community opinion leaders) as to how access to services can help adolescents [34].

## Figures and Tables

**Table 1:** Summary of findings for the effect of adolescent health interventions

Outcome	RR/SMD (95% CI)	Outcome	RR/SMD (95% CI)
Sexual and reproductive health interventions			
Mean knowledge score	SMD: 2.04 (1.31, 2.78)	Condom use	RR: 1.11 (1.04, 1.20)
Mean efficacy score	SMD: .76 (.22, 1.30)	Sexual encounter	RR: 1.00 (.93, 1.07)
Use of any contraception	RR: 1.07 (1.00, 1.14)	STI	RR: 1.08 (.79, 1.46)
Adolescent pregnancies	RR: .85 (.74, .98)	Repeat teenage pregnancies	RR: .63 (.49, .82)
Preventing female genital mutilation			
Belief that FGM/C compromise human rights of women	RR: 1.30 (.47, 3.64)	Knowledge of harmful consequences	RR: 1.53 (1.08, 2.16)
Prevalence of FGM/C	RR: .63 (.49, .82)		
Preventing dating violence			
Episodes of relationship violence	RR: .77 (.53, 1.13)	Skills related to relationship violence	SMD: 0.03 (−.11, .17)
Behavior related to relationship violence	SMD: −.07 (−.31, .16)	Knowledge related to relationship violence	SMD: .44 (.28, .60)
Promoting healthy nutrition and preventing obesity			
Mean BMI	SMD: −.08 (−.17, .01)		
Micronutrient supplementation			
Anemia	RR: .69 (.62, .76)		
Nutrition for pregnant adolescents			
Mean birth weight	RR: .25 (.08, .41)	Preterm delivery	RR: .73 (.57, .95)
Low birth weight	RR: .70 (.57, .84)	Iron-deficiency anemia	RR: .34 (.13, .89)
Serum calcium	SMD: −.17 (−.58, .23)		
Adolescent immunization			
Measles incidence	RR: .12 (.03, .38)	HPV incidence	RR: .26 (.23, .30)
Mumps incidence	RR: .96 (.42, 2.21)	HPV prevalence	RR: .56 (.38, .82)
Varicella deaths	RR: .74 (.56, .98)	HPV—vaccine coverage	RR: 1.76 (1.73, 1.80)
Meningococcal vaccine uptake	RR: 1.56 (1.45, 1.67)	HPV—CIN3 incidence	RR: .15 (.01, 2.46)
Pertussis incidence	RR: .24 (.16, .36)	HPV—vaccine uptake	RR: 1.21 (1.20, 1.23)
Rubella susceptibility	RR: .27 (.15, .46)	Multivaccine coverage	RR: 1.78 (1.41, 2.23)
HPV—incidence of genital warts	RR: .66 (.52, .84)		
Preventing substance abuse			
Smoking uptake (pure prevention)	RR: .88 [.82, .96]	Frequency of drinking days	SMD: .07 [.02, .13]
Regular smoking	RR: .59 [.42, .83]	Frequency of heavy drinking	SMD: .07 [−.01, .14]
Smoking at follow-up (smoke-free class competition)	RR: .86 [.79, .94]	Marijuana use (>12 months)	RR: .83 [.69, .99]
Lifetime smoking	RR: .73 [.64, .82]	Hard drug use (>12 months)	RR: .86 [.39, 1.90]
30-day smoking	RR: .79 [.61, 1.02]	Cannabis use	RR: .58 [.55, .62]
Alcohol consumption (quantity/week/month)	SMD: .13 [.07, .19]		
Interventions for mental health			
Knowledge of suicide prevention	SMD: .72 [.36, 1.07]	Depression	SMD: −.16 [−.26, −.05]
Anxiety	SMD: −.33 [−.59, −.06]	Knowledge of suicide	SMD: 1.51 [.57, 2.45]
Accident and injury prevention			
Incidence of injury	RR: .66 (.59, .73)	Helmet use	RR: 1.00 (.98, 1.02)
Road accidents	RR: .81 (.78, .84)	Seatbelt use	RR: .99 (.97, 1.00)
Injuries per hour of exposure	RR: .79 (.73, .86)	Injuries per number of exposure	RR: .79 (.70, .88)

BMI = body mass index; CBT = cognitive behavioral therapy; CI = confidence interval; EAT = eating attitude test; EDI = eating disorder inventory; FGM/C = female genital mutilation/cutting; HPV = human papillomavirus; RR = relative risk; SATAQ = sociocultural attitudes toward appearance questionnaire; SMD = standard mean difference; STI = sexually transmitted infections.
